# In Situ Monitoring of Drug Precipitation from Digesting Lipid Formulations Using Low-Frequency Raman Scattering Spectroscopy

**DOI:** 10.3390/pharmaceutics15071968

**Published:** 2023-07-17

**Authors:** Malinda Salim, Sara J. Fraser-Miller, Kārlis Bērziņš, Joshua J. Sutton, Keith C. Gordon, Ben J. Boyd

**Affiliations:** 1 Drug Delivery, Disposition and Dynamics, Monash Institute of Pharmaceutical Sciences, Monash University (Parkville Campus), 381 Royal Parade, Parkville, VIC 3052, Australia; malinda.salim@monash.edu; 2 *Te Whai Ao*-Dodd-Walls Centre for Photonic and Quantum Technologies, Department of Chemistry, University of Otago, Dunedin 9016, New Zealand; sara.miller@otago.ac.nz (S.J.F.-M.); karlis.berzins@sund.ku.dk (K.B.); j.sutton94@protonmail.com (J.J.S.); keith.gordon@otago.ac.nz (K.C.G.); 3 Department of Pharmacy, University of Copenhagen, Universitetsparken 2, 2100 Copenhagen, Denmark

**Keywords:** low-frequency Raman spectroscopy, drug, precipitation, in vitro digestion, lipid-based formulation, SNEDDS

## Abstract

Low-frequency Raman spectroscopy (LFRS) is a valuable tool to detect the solid state of amorphous and crystalline drugs in solid dosage forms and the transformation of drugs between different polymorphic forms. It has also been applied to track the solubilisation of solid drugs as suspensions in milk and infant formula during in vitro digestion. This study reports the use of LFRS as an approach to probe drug precipitation from a lipid-based drug delivery system (medium-chain self-nanoemulsifying drug delivery system, MC-SNEDDS) during in vitro digestion. Upon lipolysis of the digestible components in MC-SNEDDS containing fenofibrate as a model drug, sharp phonon peaks appeared at the low-frequency Raman spectral region (<200 cm^−1^), indicating the precipitation of fenofibrate in a crystalline form from the formulation. Two multivariate data analysis approaches (principal component analysis and partial least squares discriminant analysis) and one univariate analysis approach (band ratios) were explored to track these spectral changes over time. The low-frequency Raman data produces results in good agreement with in situ small angle X-ray scattering (SAXS) measurements with all data analysis approaches used, whereas the mid-frequency Raman requires the use of PLS-DA to gain similar results. This suggests that LFRS can be used as a complementary, and potentially more accessible, technique to SAXS to determine the kinetics of drug precipitation from lipid-based formulations.

## 1. Introduction

Poorly water-soluble drugs that do not dissolve in the gastrointestinal (GI) tract have limited absorption and transport into systemic circulation [1]. Enabling formulations to enhance the solubilisation of these poorly water-soluble compounds, particularly in the small intestine where most absorption occurs, are important to obtain sufficient oral bioavailability. Amongst these, lipid-based formulations such as self-nanoemulsifying drug delivery systems (SNEDDS) have been actively explored, whereby the poorly water-soluble drugs are dissolved in dispersible mixtures of lipids, surfactants, and co-solvents [2]. The process of digestion after oral administration of the formulation produced polar digestion products such as fatty acids and monoglycerides that self-assemble to form colloidal structures such as micelles, vesicles and other structures [3,4]. These structures can in some cases aid in drug solubilisation [5]; however, dilution and dispersion of the surfactants and solvents may also overall reduce the solubilisation capacity in the GI tract, inducing supersaturation that may cause the rapid re-precipitation of the drugs either in amorphous [6,7] or crystalline forms [8,9]. Similarly, the precipitation of drugs in the intestine may pose a problem for other formulation approaches such as amorphous solid dispersions [10] or drugs that have been ‘amorphised’ [11].

Understanding the extent to which the drugs would precipitate and the kinetics of drug precipitation are therefore of importance in the design and screening of formulation candidates for oral drug delivery using enabling formulations. The detection of drug precipitation from dispersed formulations has been performed by nephelometry/turbidity measurements through the dispersions or by visual observation [12], while the solid state forms of the precipitate were typically determined using crossed polarised microscopy [7,8], X-ray powder diffraction [13], differential scanning calorimetry [13], and solid-state nuclear magnetic resonance spectroscopy [14]. Meanwhile, quantitatively probing the kinetics of drug precipitation can be difficult, as it requires some form of sample separation technique. This is challenging to achieve in real time, and the timeframe for centrifugation or filtration may be similar to the kinetics of precipitation; thus, the separation process is convoluted with the kinetics one is trying to measure. Other issues may also contribute to the difficulty of extracting quantitative data for precipitation. For example, the higher density of the drug crystals should drive their sedimentation in the pellet phase during centrifugation, but the localisation of crystalline drugs at the upper lipid layer may also occur, which can lead to an erroneous interpretation of the extent of drug crystallisation [15,16]. Therefore, analytical tools that enable the in situ probing of drug precipitation in dispersions during digestion is important. One such technique that has been reported is synchrotron small angle X-ray scattering (SAXS) [9]. However, the accessibility of synchrotron-SAXS for the routine analysis of drug precipitation from oral formulations is not feasible, and as such, the development of alternative tools to complement SAXS analysis is highly desirable, particularly those that could be amenable to an industrial screening format.

**Figure 1:**
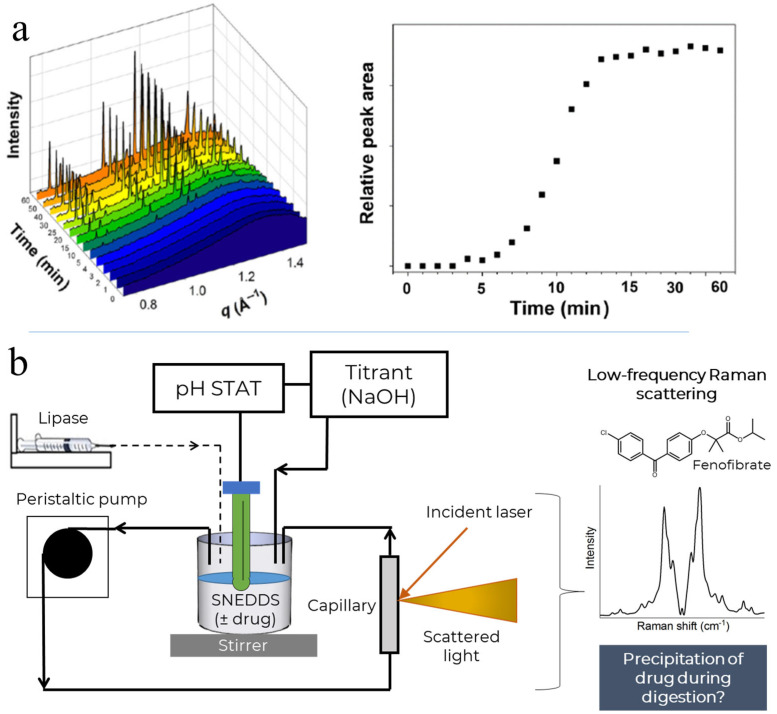
Panel (**a**)—previously published data showing in situ precipitation of fenofibrate during digestion of the same self-nanoemulsifying drug delivery system (SNEDDS) formulation as used in the current study, observed on the left as the appearance of the diffraction peaks of fenofibrate, and quantified on the right by integration of the peak at *q* = 1.15 Å^−1^ (reproduced with permission from [9]). Panel (**b**)—schematic overview of the current study depicting the in vitro digestion of the SNEDDS containing fenofibrate (unionised, logP 5.3 predicted from ChemAxon) using a pH-STAT controller. The digestion system was coupled to a low-frequency Raman spectroscopy system to assess the in situ precipitation of fenofibrate from the formulation during digestion. Part of the figure was adapted from reference [46].

In this study, we explore low-frequency Raman spectroscopy (LFRS) as a technique to probe in situ drug crystallisation in solution using a model SNEDDS formulation previously used during in situ SAXS measurements of fenofibrate from the same formulation (Figure 1 top). The analogous format was utilised (on the same pH-STAT system with the same capillary and pump) using LFRS as the analytical technique. Unlike the conventional mid to high-frequency Raman spectroscopy (typically 400–4000 cm^−1^) that primarily probes the intramolecular bond vibrations of the molecules, LFRS (<200 cm^−1^) provides signatures from both the intramolecular and intermolecular (lattice or phonon) vibrations [17,18,19,20,21,22,23,24,25]. As such, distinctive sharp phonon peaks from crystalline solids can be observed in LFRS, which can be used to study the bulk [26,27,28] and/or spatial components [29,30,31] in pharmaceutical systems. The low-frequency Raman region is particularly useful for tracking and understanding polymorphic [23,26] or solid-state transitions; examples of its use include the crystallisation of amorphous indomethacin [32], stabilisation of amorphous indomethacin with amino acids [25], transformation of caffeine under different conditions [31,33,34], and exploring dehydration processes [20,35,36]. Drug–polymer solubility [37] and the associated stabilisation effects of drug–polymer formulations [38] have also been demonstrated. Low-frequency Raman has been demonstrated as advantageous with regard to the quantification of active pharmaceutical ingredients (APIs) with examples including carbamazepine [27], griseofulvin [22], sulfathiazole polytypes [39], piroxicam [28], and caffeine-glutaric acid cocrystals [40], allowing for the detection of crystalline drugs in amorphous solid dosage forms and the determination of the solid-state polymorphic transformations [28,35,36]. Recently, LFRS in combination with principal component analysis (PCA), a dimensionality reduction tool, has also been used to characterise drug solubilisation during digestion in milk and infant formula [15,41], but its capability to detect drug crystallisation in complex suspensions in a simulated GI tract has not been widely reported, as most studies rely on mid-frequency Raman scattering [42,43]. The in situ monitoring of drug crystallisation from amorphous slurries using LFRS in the absence of digestion has however been shown for the first time by Koskela and co-workers [44]. PCA is an unsupervised dimensionality reduction tool which allows for the visualisation of a dataset based on the inherent spectral variances. This is commonly deployed with spectroscopic data where there tends to be significant co-variances. Univariate approaches can only be utilised when there are unique and distinct features in the spectroscopic data. With increasingly complex datasets, the presence of unique features becomes unobtainable. However, if available, a univariate approach is easier to implement without the need for specialist analysis software. Partial least squares discriminant analysis (PLS-DA) is a supervised multivariate analysis technique where the user defines some key variables (concentration of an ingredient or class via dummy variables) and the algorithm then finds the sources of co-variance that best describe the desired variable. This study explores the use of LFRS as an approach to probe drug precipitation from a lipid-based drug delivery system (MC-SNEDDS) during in vitro digestion, and different data analysis approaches are compared and contrasted for this application, including PCA, intensity ratios and PLS-DA [45].

## 2. Materials and Methods

Fenofibrate was purchased from AK Scientific (Union City, CA, USA). Captex 355^®^ and Capmul MCM^®^ were from Abitec Corporation (Janesville, WI, USA). Cremophor EL^®^ was obtained from BASF Corporation (Washington, NJ, USA). Trizma^®^ maleate (reagent grade) was from Sigma-Aldrich (St. Louis, MO, USA). Calcium chloride dihydrate (>99%) was from Ajax Finechem (Seven Hills, NSW, Australia). Sodium chloride (>99%) was purchased from Chem Supply (Gillman, SA, Australia). USP grade pancreatin extract was purchased from Southern Biologicals (Nunawading, VIC, Australia).

The model poorly water-soluble drug (fenofibrate) was loaded into a medium-chain SNEDDS formulation (MC-SNEDDS) at 200% drug solubility as described previously [9]. The MC-SNEDDS contains mixtures of Captex^®^, Capmul MCM^®^, Cremophor EL^®^ (each 0.2 g) and ethanol (0.07 g) into which fenofibrate (192 mg) was added, vortexed and incubated at 70 °C for 5 hrs. The formulation was subsequently placed on a roller mixer in a 40 °C oven overnight. Tris buffer (20 mL) was added to the formulation, which dispersed to form a translucent nanoemulsion system, and the sample was transferred into a jacketed glass digestion vessel (82 mm height, 78 mm OD, 5–70 mL, Metrohm) maintained at 37 °C. The buffer contained 50 mM Trizma^®^ maleate, 150 mM sodium chloride and 5 mM calcium chloride dihydrate at pH 6.5.

Using a peristaltic pump (Masterflex™ C/L Variable-Speed Tubing Pump), the dispersed sample was circulated at approximately 10 mL/min through silicone tubing (2 mm ID, 4 mm OD) into a static quartz capillary (1.5 mm OD, 80 mm length, 0.01 mm wall thickness) that sits in the laser beam path (Figure 1b). The total volume of solution in circulation was about 2.5 mL at any point in time. Configuration of the LFRS setup has been described previously [15,41] where a 785 nm laser source (Ondax, Inc. Monrovia, CA, USA) was focussed onto the quartz capillary containing the sample and the spectrum was collected at a 135° angle relative to the collecting lens with a 0.5 s acquisition time × 60 accumulations for 90 frames. Pancreatic lipase (2.25 mL of 0.217 g freeze-dried pancreatin/mL tris buffer; activity about 700 tributyrin unit) was added into the digestion vessel (sample stirred at speed setting 2, approximately 250 rotation per minute) to initiate lipid digestion after 2 min of circulating the dispersion. Titration was performed using a Metrohm titrator (902 Titrando) and 2 M NaOH titrant. The spectral acquisition for blank MC-SNEDDS containing no fenofibrate was also collected to discern signals between the drug and the digesting formulation.

Principal component analysis (PCA) of the low-frequency Raman data was performed using the data mining software Orange [47] version 3.22.0 (University of Ljubljana, Ljubljana, Slovenia). The spectra were preprocessed with a linear baseline correction (from −250 to 250 cm^−1^), and vector normalisation (from 8–200 cm^−1^) was applied. For the mid-frequency region, the spectra were preprocessed with a linear baseline correction (from 500–1800 cm^−1^) and vector normalisation (from 1000–1800 cm^−1^). The principal component that corresponded to the drug signal was monitored as a function of time to reveal changes occurring during dispersion and digestion.

**Figure 2:**
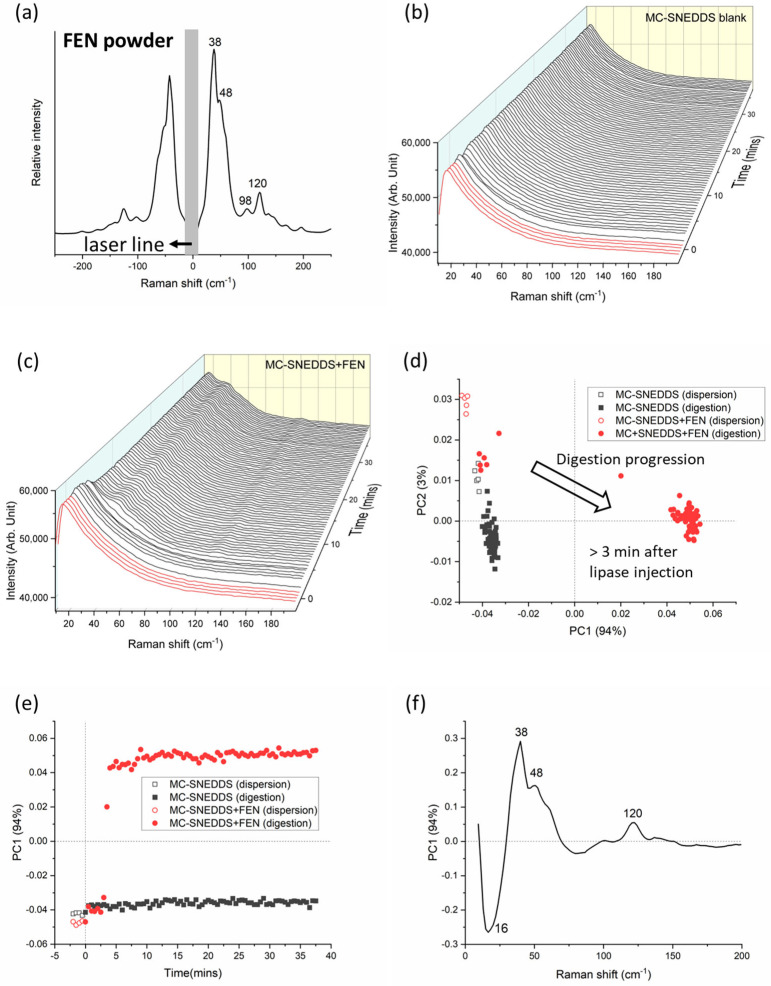
(**a**) Characteristic Raman spectrum for fenofibrate (FEN) at the low-frequency region. (**b**) Raman spectra of medium-chain self-nanoemulsifying drug delivery system (MC-SNEDDS) during dispersion (red lines, time < 0 min) and digestion (black lines, time > 0 min) containing no fenofibrate and (**c**) with fenofibrate. Lipase was injected at 0 min to initiate digestion. (**d**) Principal component analysis (PCA) of the formulations (MC-SNEDDS and MC-SNEDDS+FEN) during dispersion and digestion as a two-dimensional scores plot of the first two principal components (97% explained variance) based on low-frequency region (8–200 cm^−1^) spectra; and (**e**) changes in the first principal component (PC1) as a function of dispersion and digestion time. (**f**) The corresponding loadings plot for PC1. The loadings plot for PC2 was shown as Figure S1 in the Supplementary Materials.

Band ratios were carried out based on the band intensity at each wavenumber after linear baseline correction in Orange [47] version 3.22.0 (−200 to 200 cm^−1^ in the low-frequency region and from 1000 to 1800 cm^−1^ in the mid-frequency region). The intensity ratio for the low-frequency region (I_38_/I_16_) utilised the intensities at the pixels representing 38 and 16 cm^−1^. The 38 cm^−1^ mode is associated with the crystalline form of the drug, whereas the feature at 16 cm^−1^ is attributed to the MC-SNEDDS formulation or medium. This feature is observed even when fenofibrate is absent from the mixture (Figure 2d). The intensity ratio for the mid-frequency region (I_1648_/I_1657_) utilised the signal intensities at pixels representing 1648 and 1657 cm^−1^. The 1648 cm^−1^ band is associated with crystalline fenofibrate and is observed after digestion in which the drug is now suspended in the medium in the crystalline state. The less intense feature at 1657 cm^−1^ is also attributed to fenofibrate, but the band is shifted when the fenofibrate is solubilised in the MC-SNEDDS formulation.

Partial least squares-discriminant analysis (PLS-DA) was performed using Unscrambler X V 10.5 (Camo, Oslo, Norway) after spectral preprocessing. The low-frequency Raman spectra were preprocessed using standard normal variate (SNV) from 10 to 200 cm^−1^. The mid-frequency Raman spectra first underwent linear baseline correction from 1000 to 1800 cm^−1^ followed by SNV of the same region. The first 3 data points (dispersion phase) of the MC-SNEDDS and MC-SNEDDS + FEN samples along with the last three data points (digestion phase) from the MC-SNEDDS samples were given the dummy variable 0. The final 3 points of the MC-SNEDDS + FEN sample were given the dummy variable 1. A PLS regression model was then developed using these time points for each spectral region using the Unscrambler X V 10.5 Kernel algorithm with full cross-validation. All samples and time points were then predicted using this model.

## 3. Results

The LFRS spectrum for fenofibrate powder displayed peaks in the low-frequency regime at approximately 38 and 120 cm^−1^ (see Figure 2a). At approximately 3 min after the initiation of digestion, there were changes in the low-frequency Raman spectra of MC-SNEDDS during dispersion and digestion without and with added fenofibrate; see Figure 2b,c respectively. Specifically, the appearance of peaks in Figure 2c at approximately 38 and 120 cm^−1^ characteristic of the drug signals occurred approximately 3 min after the initiation of digestion, indicating drug precipitation. Appearance of the sharp Raman scattering peaks at this low wavenumber region suggests that the precipitated fenofibrate was in the crystalline form. Fenofibrate is a non-ionisable drug, so it is less likely to interact with lipid digestion products to form amorphous lipophilic salt forms of the drug, unlike weakly basic drugs. Therefore, it was not unexpected that on this basis (and the previous studies in Figure 1a), the fenofibrate precipitated in a crystalline form.

Rapid precipitation of the fenofibrate can also be clearly discerned from multivariate analysis of the Raman spectra (Figure 2d,e) where changes in the first principal component (PC1), which correspond to the fenofibrate signal, in a positive PC1 scores space, were observed shortly after lipase injection. Assuming a 0% and 100% extent of drug precipitation at the start of dispersion and the end of digestion, respectively, the relative PC1 score at 3 min (where fenofibrate peaks started to appear) was approximately 15%, which may suggest that fenofibrate could be detected at a concentration of about 1.4 mg/mL. However, further studies are required to confirm the limit of detection of fenofibrate in MC-SNEDDS, since PCA is, in nature, a non-quantitative approach. The loadings plot in Figure 2f shows the main positive features contributing to these changes which correlate with the peaks from fenofibrate powder in Figure 2a. Thus, both the observation of the raw data and the PCA results support the findings from the previous SAXS studies where the onset of drug precipitation (determined by the appearance of Bragg peaks in in situ SAXS profiles) was 4 min after the injection of lipase into the fenofibrate-loaded MC-SNEDDS, which corresponded to approximately 50% digestion of the formulation. Such findings indicate a loss of drug solubilising capacity causing drug precipitation [9]. The generation of more polar medium-chain digestion products after the digestion of a medium-chain triglycerides-based SNEDDS formulation also resulted in the precipitation of danazol in vitro, which also correlated with reduced bioavailability in vivo [48].

**Figure 3:**
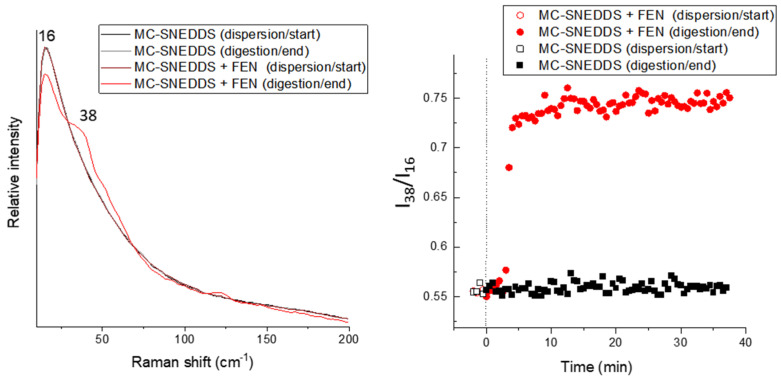
Low-frequency Raman band ratio-based assessment of the precipitation of fenofibrate (FEN) during digestion. The low-frequency Raman spectra and associated bands selected for the ratio I_38_/I_16_ are shown and the left, and the associated ratios over time are shown on the right.

A univariate approach was also explored as it is often perceived as more accessible than multivariate approaches. The bands were selected based on the PCA loadings positions, which have a significant contribution to PC1 separation with the band at 16 cm^−1^ present in all formulations associated with the MC-SNEDDS formulation or medium and the band at 38 cm^−1^ attributed to the crystalline drug. The ratio (I_38_/I_16_) of the band intensities at 38 and 16 cm^−1^ over time are shown in Figure 3 (right panel) and are consistent with those observed with the PCA. This suggests that a univariate approach to look at the low-frequency Raman changes over time is applicable for tracking drug precipitation during digestion in this example.

**Figure 4:**
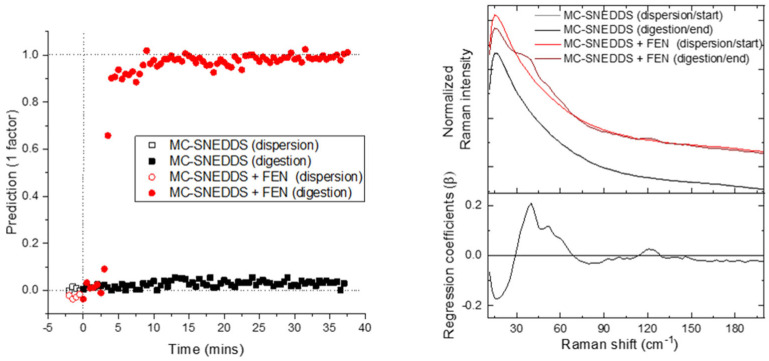
PLS-DA predictions using the low-frequency Raman data. On the left is the prediction versus time; and on the right is the associated weighted regression coefficients in comparison to the average start and end spectra for each run.

PLS-DA was also explored as a user-defined multivariate model. The PLS-DA of the low-frequency Raman data was almost identical to that of the PCA for the prediction/PC1 scores values versus time and associated regression coefficients/PC1 loadings plots (Figure 2 and Figure 4). This highlights that the major source of variance in the low-frequency Raman region is associated with the precipitation during digestion.

**Figure 5:**
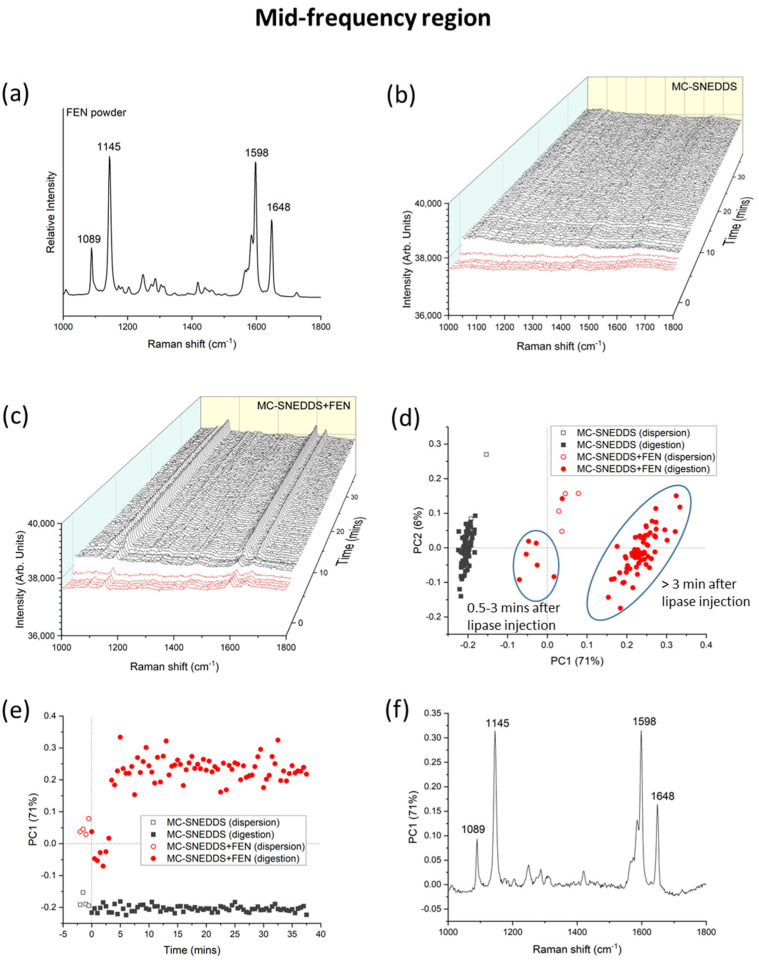
(**a**) Characteristic Raman spectrum for fenofibrate (FEN) at the mid-frequency region. (**b**) Mid-frequency Raman spectra of medium-chain self-nanoemulsifying drug delivery system (MC-SNEDDS) during dispersion (red lines, time < 0 min) and digestion (black lines, time > 0 min) containing no fenofibrate and (**c**) with fenofibrate. Lipase was injected at 0 min to initiate digestion. (**d**) Principal component analysis (PCA) of the formulations (MC-SNEDDS and MC-SNEDDS+FEN) during dispersion and digestion based on the mid-frequency region (from 1000 to 1800 cm^−1^) spectra. (**e**) Changes in the first principal component (PC1) as a function of dispersion and digestion time and (**f**) the corresponding loadings plot for PC1.

Separate analysis of the mid-frequency Raman spectral region also showed an increase in peak intensity for fenofibrate as digestion of the MC-SNEDDS formulation was initiated (Figure 5). The PC1 kinetic profile in Figure 5e illustrates the rapid change occurring during digestion, while comparison of the loadings plot in Figure 5f with that for the fenofibrate powder in Figure 5a is consistent with the presence of solid crystalline fenofibrate. Figure 5d also discriminates the blank formulations from drug-loaded formulations and dispersion from digestion with respect to changes in the main features of the Raman spectra. However, unlike the low-frequency region, some fenofibrate peaks were present at the initial time points during dispersion (Figure 5c). This may be attributed to detection of the drug solubilised in the SNEDDS, as the mid-frequency Raman bands are characteristic of intramolecular vibrations and will be present in both the crystalline and solubilised form albeit with some subtle differences in the relative intensities and positions (Figure S2).

**Figure 6:**
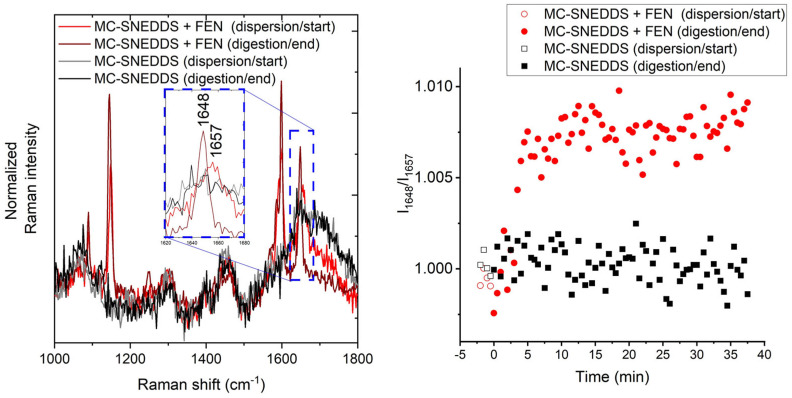
Mid-frequency Raman band ratio-based assessment of the precipitation of fenofibrate (FEN) during digestion. The mid-frequency Raman spectra and associated bands selected for the ratio I_1648_/I_1657_ are shown on the left, and the associated ratios over time are shown on the right.

Univariate analysis is more challenging within the mid-frequency Raman range due to the overlapping nature of the drug bands present in the dissolved and crystalline states. The band around 1650 cm^−1^ (C=O stretch) [49] has the biggest shift and change of shape observed in this dataset and is the target for this univariate approach. The broad band centred at 1657 cm^−1^ is attributed to the dissolved drug, and the sharper version at 1648 cm^−1^ is attributed to the crystalline form. The intensity ratio I_1648_/I_1657_ was used, and the resulting ratio over time is shown in Figure 6. This approach gives a start position in the same location for the blank and fenofibrate-loaded runs. This is similar to what is observed in the low-frequency Raman data but different to that demonstrated with the PCA of the mid-frequency Raman data where the drug-loaded sample pre-digestion was intermediate to the blank sample and digested time points. The data are clearly noisier than that of the LFR equivalent, which is perhaps unsurprising due to the noisier nature of these data compared to the LFR region and the relatively close proximity and slight overlap of the bands used in this ratio.

**Figure 7:**
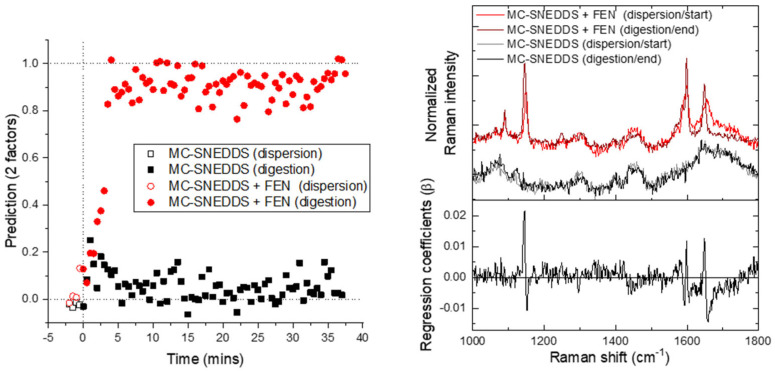
PLS-DA predictions using the mid-frequency Raman data. On the left is the prediction versus time; and on the right is the associated weighted regression coefficients in comparison to the average start and end spectra for each run.

The PLS-DA of the mid-frequency region (Figure 7) generated results that were easier to interpret than the analogous PCA with the dispersion phase of both runs having similar dummy variables. The precipitation of fenofibrate during digestion was clearly shown distinct to the digestion of SNEDDS alone. The associated PLS regression coefficients showed a subtle differentiation between the precipitated and dissolved forms of the drug in the form of band shift and bandwidth changes (Figure 7, right). Of the mid-frequency Raman analysis methods, PLS-DA was the only method trialled that gave similar results to SAXS and low-frequency Raman techniques. This highlights that a user-defined approach such as PLS-DA can at times be more applicable, particularly when the differences between the dissolved and crystalline drug are subtle.

These observations collectively suggest that both low- and mid-frequency Raman bands could be used to track in situ drug precipitation during the digestion of lipid-based formulations, and a direct response could be observed in the former due to the intramolecular nature of these bands. A low-frequency Raman approach can generate data that can be used using a simple univariate approach; however, the mid-frequency Raman region can be analysed in a manner (e.g., PLS-DA) to pull out similar information, albeit slightly noisier. This confirms that LFRS is a valuable analytical tool to determine the kinetics of drug precipitation without the need for either a synchrotron X-ray source nor the need to “separate and sample” precipitated drug and handle the challenges associated with kinetic resolution therein.

## 4. Conclusions

Successful formulations for the oral delivery of poorly-water soluble drugs are intended to keep the drug in solution during dispersion and digestion in the GI tract, as precipitation of the drugs in a less soluble crystalline form prior to absorption can reduce the oral bioavailability of the drug. New techniques enabling the detection of drug crystallisation in situ during the digestion of lipid formulations are therefore valuable. In this study, we showed that LFRS can be used to measure the kinetics of precipitation of drug during the digestion of a lipid-based formulation (in this case, a MC-SNEDDS loaded with fenofibrate as a model case study). Precipitation of the fenofibrate was rapid with significant drug crystals observed 3 min into digestion. Our results support previous findings from SAXS, suggesting that LFRS can be used as an alternative and more accessible approach to screen drug precipitation from enabling lipid formulations during digestion. The inherent capability of LFRS to detect drug crystallisation in solution in situ also meant that the technique could necessitate the development of other amorpho us (non-lipid-based) formulations such as amorphous solid dispersions, where the stability of the drug as an amorphous form remained challenging due to the tendency of the drug to re-crystallise both during storage and upon contact with an aqueous medium. In addition, the technique could also be potentially used to inform whether changes to the polymorphic forms of the drug post re-crystallisation have occurred. In this proof-of-concept study, fenofibrate precipitated as the same polymorph in MC-SNEDDS as the crystalline starting material, but in more complex systems, the appearance of multiple solid-state transformations can occur during dispersion and digestion. Hence, looking forward, it is anticipated that LFRS could be used as an accessible tool to probe the kinetics of drug crystallisation with simultaneous polymorphic screening in situ.

## Data Availability

Data is contained within the article or Supplementary Materials.

## Supplementary Materials

The following supporting information can be downloaded at: https://www.mdpi.com/article/10.3390/pharmaceutics15071968/s1, Figure S1: Loadings plot for PC2 based on the low-frequency region (8–200 cm^−1^); Figure S2: Mid-frequency Raman spectra of medium-chain self-nanoemulsifying drug delivery system (MC-SNEDDS) before digestion (2 min prior to lipase injection) and after digestion.

## References

[1] Hauss D.J. (2007). Oral Lipid-Based Formulations: Enhancing the Bioavailability of Poorly Water-Soluble Drugs. Drugs and the Pharmaceutical Sciences.

[2] Siqueira S.D., Müllertz A., Gräeser K., Kasten G., Mu H., Rades T. (2017). Influence of drug load and physical form of cinnarizine in new SNEDDS dosing regimens: In vivo and in vitro evaluations. AAPS J..

[3] Fatouros D.G., Deen G.R., Arleth L., Bergenstahl B., Nielsen F.S., Pedersen J.S., Mullertz A. (2007). Structural development of self nano emulsifying drug delivery systems (SNEDDS) during in vitro lipid digestion monitored by small-angle X-ray scattering. Pharm. Res..

[4] Warren D.B., Anby M.U., Hawley A., Boyd B.J. (2011). Real Time Evolution of Liquid Crystalline Nanostructure during the Digestion of Formulation Lipids Using Synchrotron Small-Angle X-ray Scattering. Langmuir.

[5] Boyd B.J., Salim M., Clulow A.J., Ramirez G., Pham A.C., Hawley A. (2018). The impact of digestion is essential to the understanding of milk as a drug delivery system for poorly water soluble drugs. J. Control. Release.

[6] Thomas N., Holm R., Garmer M., Karlsson J.J., Müllertz A., Rades T. (2013). Supersaturated self-nanoemulsifying drug delivery systems (Super-SNEDDS) enhance the bioavailability of the poorly water-soluble drug simvastatin in dogs. AAPS J..

[7] Sassene P.J., Knopp M.M., Hesselkilde J.Z., Koradia V., Larsen A., Rades T., Müllertz A. (2010). Precipitation of a Poorly Soluble Model Drug during In Vitro Lipolysis: Characterization and Dissolution of the Precipitate. J. Pharm. Sci..

[8] Thomas N., Richter K., Pedersen T.B., Holm R., Müllertz A., Rades T. (2014). In vitro lipolysis data does not adequately predict the in vivo performance of lipid-based drug delivery systems containing fenofibrate. AAPS J..

[9] Khan J., Hawley A., Rades T., Boyd B.J. (2016). In Situ Lipolysis and Synchrotron Small-Angle X-ray Scattering for the Direct Determination of the Precipitation and Solid-State Form of a Poorly Water-Soluble Drug During Digestion of a Lipid-Based Formulation. J. Pharm. Sci..

[10] Alonzo D.E., Gao Y., Zhou D., Mo H., Zhang G.G., Taylor L.S. (2011). Dissolution and precipitation behavior of amorphous solid dispersions. J. Pharm. Sci..

[11] Dengale S.J., Grohganz H., Rades T., Löbmann K. (2016). Recent advances in co-amorphous drug formulations. Adv. Drug Deliv. Rev..

[12] Baloch J., Sohail M.F., Sarwar H.S., Kiani M.H., Khan G.M., Jahan S., Rafay M., Chaudhry M.T., Yasinzai M., Shahnaz G. (2019). Self-Nanoemulsifying Drug Delivery System (SNEDDS) for Improved Oral Bioavailability of Chlorpromazine: In Vitro and In Vivo Evaluation. Medicina.

[13] Bhattacharya S., Brittain H.G., Suryanarayanan R. (2018). Thermoanalytical and crystallographic methods. Polymorphism in Pharmaceutical Solids.

[14] Tishmack P.A. (2018). Solid-State Nuclear Magnetic Resonance Spectroscopy. Polymorphism in Pharmaceutical Solids.

[15] Salim M., Fraser-Miller S.J., Bērziņš K., Sutton J.J., Ramirez G., Clulow A.J., Hawley A., Beilles S., Gordon K.C., Boyd B.J. (2020). Low-Frequency Raman Scattering Spectroscopy as an Accessible Approach to Understand Drug Solubilization in Milk-Based Formulations during Digestion. Mol. Pharm..

[16] Salim M., Ramirez G., Clulow A.J., Hawley A., Boyd B.J. (2023). Implications of the Digestion of Milk-Based Formulations for the Solubilization of Lopinavir/Ritonavir in a Combination Therapy. Mol. Pharm..

[17] Bērziņš K., Fraser-Miller S.J., Rades T., Gordon K.C. (2022). Low-Frequency Raman Spectroscopy as an Avenue to Determine the Transition Temperature of β- and γ-Relaxation in Pharmaceutical Glasses. Anal. Chem..

[18] Bērziņš K., Sales R.E., Barnsley J.E., Walker G., Fraser-Miller S.J., Gordon K.C. (2020). Low-wavenumber Raman spectral database of pharmaceutical excipients. Vib. Spectrosc..

[19] Bērziņš K., Fraser-Miller S.J., Rades T., Gordon K.C. (2019). Low-Frequency Raman Spectroscopic Study on Compression-Induced Destabilization in Melt-Quenched Amorphous Celecoxib. Mol. Pharm..

[20] Robert C., Fraser-Miller S.J., Bērziņš K., Okeyo P.O., Rantanen J., Rades T., Gordon K.C. (2021). Monitoring the Isothermal Dehydration of Crystalline Hydrates Using Low-Frequency Raman Spectroscopy. Mol. Pharm..

[21] Bērziņš K., Fraser-Miller S.J., Gordon K.C. (2021). Recent advances in low-frequency Raman spectroscopy for pharmaceutical applications. Int. J. Pharm..

[22] Mah P.T., Fraser S.J., Reish M.E., Rades T., Gordon K.C., Strachan C.J. (2015). Use of low-frequency Raman spectroscopy and chemometrics for the quantification of crystallinity in amorphous griseofulvin tablets. Vib. Spectrosc..

[23] Larkin P.J., Dabros M., Sarsfield B., Chan E., Carriere J.T., Smith B.C. (2014). Polymorph Characterization of Active Pharmaceutical Ingredients (APIs) Using Low-Frequency Raman Spectroscopy. Appl. Spectrosc..

[24] Larkin P.J., Wasylyk J., Raglione M. (2015). Application of Low- and Mid-Frequency Raman Spectroscopy to Characterize the Amorphous-Crystalline Transformation of Indomethacin. Appl. Spectrosc..

[25] Walker G., Römann P., Poller B., Löbmann K., Grohganz H., Rooney J.S., Huff G.S., Smith G.P.S., Rades T., Gordon K.C. (2017). Probing Pharmaceutical Mixtures during Milling: The Potency of Low-Frequency Raman Spectroscopy in Identifying Disorder. Mol. Pharm..

[26] Hédoux A., Guinet Y., Derollez P., Dudognon E., Correia N.T. (2011). Raman spectroscopy of racemic ibuprofen: Evidence of molecular disorder in phase II. Int. J. Pharm..

[27] Inoue M., Hisada H., Koide T., Fukami T., Roy A., Carriere J., Heyler R. (2019). Transmission low-frequency Raman spectroscopy for quantification of crystalline polymorphs in pharmaceutical tablets. Anal. Chem..

[28] Lipiäinen T., Fraser-Miller S.J., Gordon K.C., Strachan C.J. (2018). Direct comparison of low- and mid-frequency Raman spectroscopy for quantitative solid-state pharmaceutical analysis. J. Pharm. Biomed. Anal..

[29] Hisada H., Inoue M., Koide T., Carriere J., Heyler R., Fukami T. (2015). Direct high-resolution imaging of crystalline components in pharmaceutical dosage forms using low-frequency Raman spectroscopy. Org. Process Res. Dev..

[30] Bērziņš K., Fraser-Miller S.J., Gordon K.C. (2021). Pseudo-3D Subsurface Imaging of Pharmaceutical Solid Dosage Forms Using Micro-spatially Offset Low-Frequency Raman Spectroscopy. Anal. Chem..

[31] Hubert S., Briancon S., Hedoux A., Guinet Y., Paccou L., Fessi H., Puel F. (2011). Process induced transformations during tablet manufacturing: Phase transition analysis of caffeine using DSC and low frequency micro-Raman spectroscopy. Int. J. Pharm..

[32] Hédoux A., Paccou L., Guinet Y., Willart J.-F., Descamps M. (2009). Using the low-frequency Raman spectroscopy to analyze the crystallization of amorphous indomethacin. Eur. J. Pharm. Sci..

[33] Hédoux A., Guinet Y., Paccou L., Danède F., Derollez P. (2013). Polymorphic transformation of anhydrous caffeine upon grinding and hydrostatic pressurizing analyzed by low-frequency raman spectroscopy. J. Pharm. Sci..

[34] Hédoux A., Decroix A.-A., Guinet Y., Paccou L., Derollez P., Descamps M. (2011). Low-and high-frequency Raman investigations on caffeine: Polymorphism, disorder and phase transformation. J. Phys. Chem. B.

[35] Remoto P.I.J.G., Bērziņš K., Fraser-Miller S.J., Korter T.M., Rades T., Rantanen J., Gordon K.C. (2023). Exploring the Solid-State Landscape of Carbamazepine during Dehydration: A Low Frequency Raman Spectroscopy Perspective. Pharmaceutics.

[36] Remoto P.J.G., Bērziņš K., Fraser-Miller S.J., Korter T.M., Rades T., Rantanen J., Gordon K.C. (2022). Elucidating the Dehydration Mechanism of Nitrofurantoin Monohydrate II Using Low-Frequency Raman Spectroscopy. Cryst. Growth Des..

[37] Latreche M., Willart J.-F., Paccou L., Guinet Y., Danède F., Hédoux A. (2020). Contribution of low-frequency Raman spectroscopy to determine the solubility curves of drugs in polymers: The case of paracetamol/PVP. Eur. J. Pharm. Biopharm..

[38] Bērziņš K., Fraser-Miller S.J., Walker G.F., Rades T., Gordon K.C. (2021). Investigation on formulation strategies to mitigate compression-induced destabilization in supersaturated celecoxib amorphous solid dispersions. Mol. Pharm..

[39] Iwata K., Karashima M., Ikeda Y., Inoue M., Fukami T. (2018). Discrimination and quantification of sulfathiazole polytypes using low-frequency Raman spectroscopy. CrystEngComm.

[40] Inoue M., Osada T., Hisada H., Koide T., Fukami T., Roy A., Carriere J., Heyler R. (2019). Solid-state quantification of cocrystals in pharmaceutical tablets using transmission low-frequency Raman spectroscopy. Anal. Chem..

[41] Salim M., Fraser-Miller S.J., Sutton J.J., Bērziņš K., Hawley A., Clulow A.J., Beilles S., Gordon K.C., Boyd B.J. (2019). Application of Low-Frequency Raman Scattering Spectroscopy to Probe in Situ Drug Solubilization in Milk during Digestion. J. Phys. Chem. Lett..

[42] Stillhart C., Imanidis G., Kuentz M. (2013). Insights into Drug Precipitation Kinetics during In Vitro Digestion of a Lipid-Based Drug Delivery System Using In-Line Raman Spectroscopy and Mathematical Modeling. Pharm. Res..

[43] Alskär L.C., Keemink J., Johannesson J., Porter C.J.H., Bergström C.A.S. (2018). Impact of Drug Physicochemical Properties on Lipolysis-Triggered Drug Supersaturation and Precipitation from Lipid-Based Formulations. Mol. Pharm..

[44] Koskela J., Sutton J.J., Lipiäinen T., Gordon K.C., Strachan C.J., Fraser-Miller S.J. (2022). Low- versus Mid-frequency Raman Spectroscopy for in Situ Analysis of Crystallization in Slurries. Mol. Pharm..

[45] Smith G.P.S., McGoverin C.M., Fraser S.J., Gordon K.C. (2015). Raman imaging of drug delivery systems. Adv. Drug Deliv. Rev..

[46] Salim M., Eason T., Boyd B.J. (2022). Opportunities for milk and milk-related systems as ‘new’ low-cost excipient drug delivery materials. Adv. Drug Deliv. Rev..

[47] Demšar J., Curk T., Erjavec A., Gorup Č., Hočevar T., Milutinovič M., Možina M., Polajnar M., Toplak M., Starič A. (2013). Orange: Data mining toolbox in Python. J. Mach. Learn. Res..

[48] Porter C.J.H., Kaukonen A.M., Boyd B.J., Edwards G.A., Charman W.N. (2004). Susceptibility to lipase-mediated digestion reduces the oral bioavailability of danazol after administration as a medium-chain lipid-based microemulsion formulation. Pharm. Res..

[49] Heinz A., Gordon K.C., McGoverin C.M., Rades T., Strachan C.J. (2009). Understanding the solid-state forms of fenofibrate—A spectroscopic and computational study. Eur. J. Pharm. Biopharm..

