# Supplementary material for: In Situ Monitoring of Drug Precipitation from Digesting Lipid Formulations Using Low-Frequency Raman Scattering Spectroscopy
Source: Pharmaceutics. 2023 Jul 17;15(7):1968. doi: 10.3390/pharmaceutics15071968 (PMC10383805; doi:10.3390/pharmaceutics15071968)
Supplement: Supplementary file 1 [file pharmaceutics-15-01968-s001.zip › pharmaceutics-2454376-supplementary.pdf]

In Situ Monitoring of Drug Precipitation from Digesting Lipid Formulations Using Low-Frequency Raman Scattering Spectroscopy

Malinda Salim [1,†], Sara J. Fraser-Miller [2,†], Kārlis Bērziņš [2,3], Joshua J. Sutton [2], Keith C. Gordon [2] and Ben J. Boyd [1,3,*]

[1] Drug Delivery, Disposition and Dynamics, Monash Institute of Pharmaceutical Sciences, Monash University (Parkville Campus), 381 Royal Parade, Parkville, VIC 3052, Australia; malinda.salim@monash.edu

[2] *Te Whai Ao*-Dodd-Walls Centre for Photonic and Quantum Technologies, Department of Chemistry, University of Otago, Dunedin 9016, New Zealand; sara.miller@otago.ac.nz (S.J.F.-M.); karlis.berzins@sund.ku.dk (K.B.); j.sutton94@protonmail.com (J.J.S.); keith.gordon@otago.ac.nz (K.C.G.)

[3] Department of Pharmacy, University of Copenhagen, Universitetsparken 2, 2100 Copenhagen, Denmark

* Correspondence: ben.boyd@monash.edu; Tel.: +61-3-99039112; Fax: +61-3-99039583

† These authors contributed equally to this work.

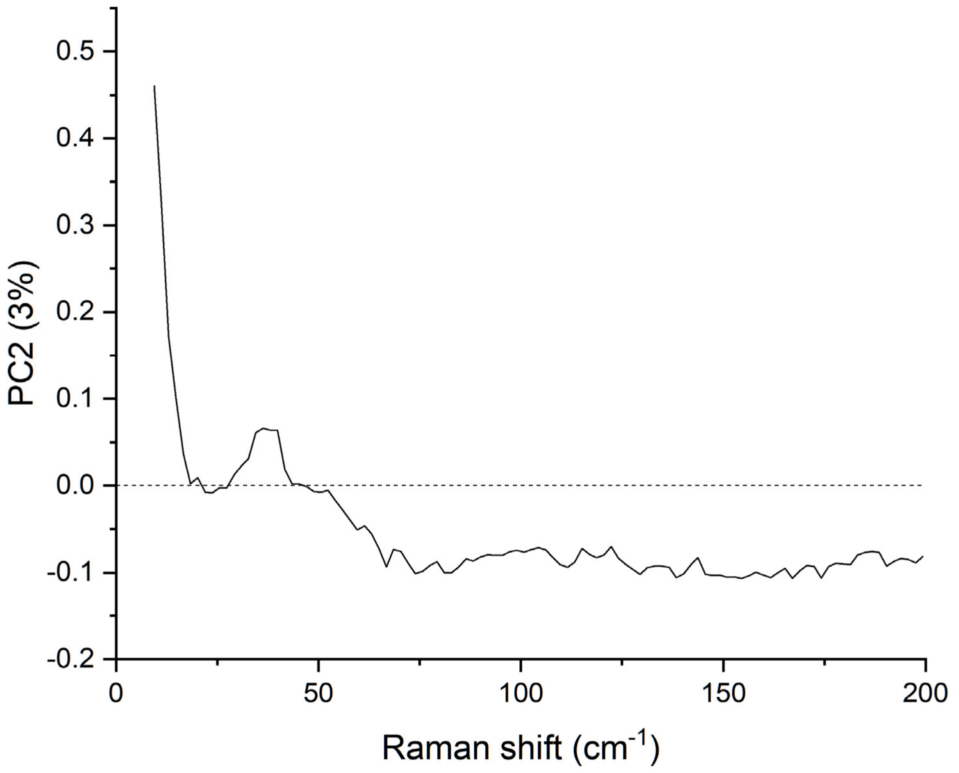

Figure S1. Loadings plot for PC2 based on the low-frequency region (8-200 cm^{-1}).

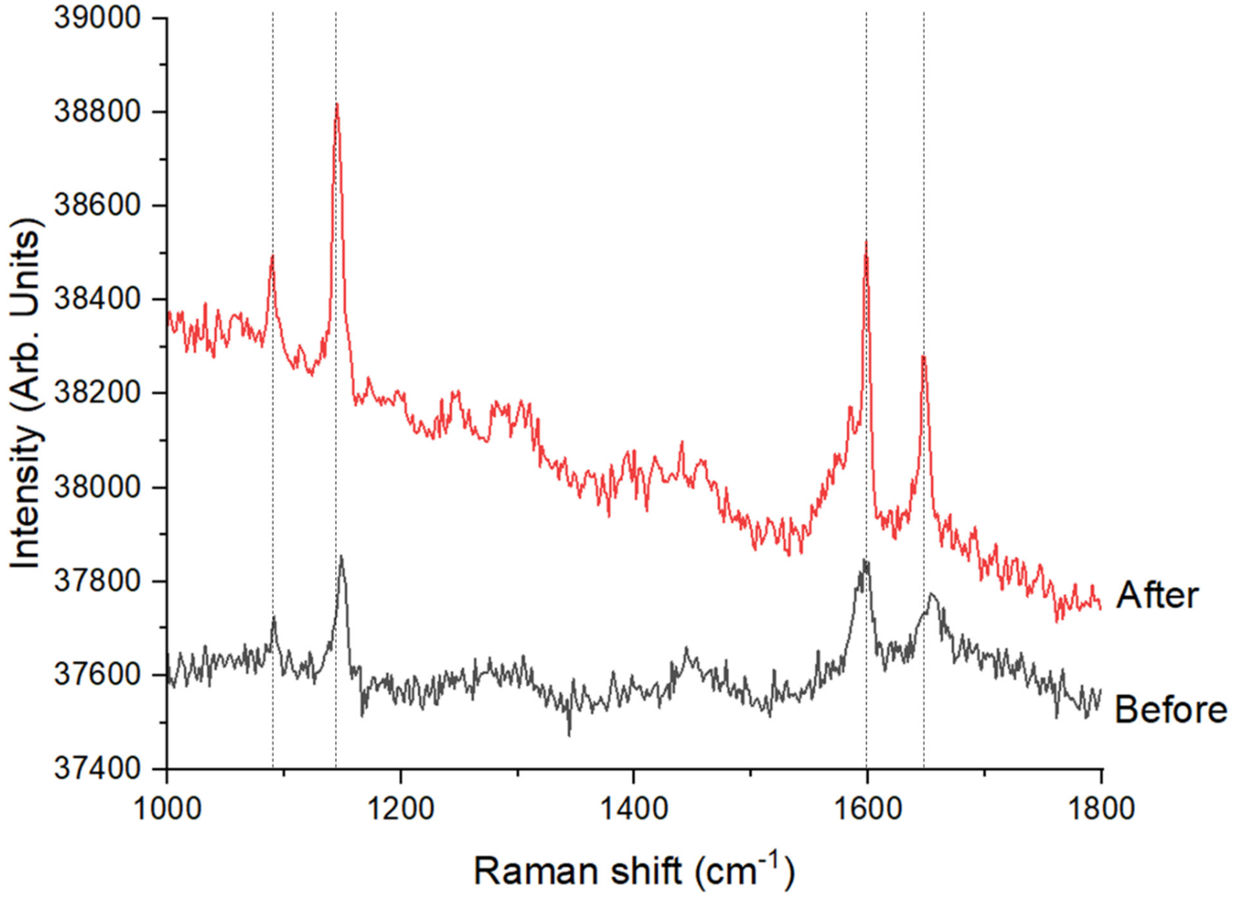

Figure S2. Mid-frequency Raman spectra of medium chain self-nanoemulsifying drug delivery system (MC-SNEDDS) before digestion (2 mins prior to lipase injection) and after digestion.
